# Age-related genomic alterations and chemotherapy sensitivity in osteosarcoma: insights from cancer genome profiling analyses

**DOI:** 10.1007/s10147-024-02673-2

**Published:** 2024-12-17

**Authors:** Hidetatsu Outani, Masachika Ikegami, Yoshinori Imura, Sho Nakai, Haruna Takami, Yuki Kotani, Akitomo Inoue, Seiji Okada

**Affiliations:** 1 Department of Orthopaedic Surgery, Osaka University Graduate School of Medicine, 2-2 Yamadaoka, Suita City, Osaka Japan; 2 Department of Musculoskeletal Oncology, Tokyo Metropolitan Cancer and Infection Diseases Center, Komagome Hospital, 3-18-22 Honkomagome, Bunkyo-ku, Tokyo, Japan; 3 Division of Cellular Signaling, National Cancer Center Research Institute, 5-1-1 Tsukiji, Chuo-ku, Tokyo, Japan

**Keywords:** Osteosarcoma, Cancer genome profiling, Chemotherapy, Chemotherapy sensitivity, Gene alteration

## Abstract

**Background:**

Osteosarcoma, the most common primary bone malignancy, has a complex genetic basis and two incidence peaks. In younger patients, the standard treatment involves wide surgical resection combined with adjuvant chemotherapy; however, the role of chemotherapy in elderly patients remains controversial. The aims of this study were to investigate genetic differences between younger and elderly patients with osteosarcoma and to identify genetic signatures associated with chemotherapy response.

**Methods:**

Genetic alterations were analyzed using cancer genome profiling data for 204 patients with osteosarcoma obtained from the Center for Cancer Genomics and Advanced Therapeutics.

**Results:**

The mutation spectrum was consistent with previous results for osteosarcoma. *CCNE1*, *MCL1*, *MYC*, and *RB1* alterations were significantly associated with a younger age, while *CDK4*, *CDKN2A*, *CDKN2B*, *H3F3A*, *KMT2D*, *MDM2*, *RAC1*, and *SETD2* alterations were significantly associated with an older age. Age, unsupervised clustering of gene alterations, and *MYC* amplifications were significantly associated with the response to ifosfamide. Notably, both clustered mutation signatures and *MYC* amplification were correlated with age.

**Conclusions:**

These findings suggest that distinct oncogenic mechanisms contribute to differential sensitivity to chemotherapy in younger and elderly patients. Cancer genome profiling may aid in chemotherapy selection, and its early implementation is recommended to optimize treatment strategies.

**Supplementary Information:**

The online version contains supplementary material available at 10.1007/s10147-024-02673-2.

## Introduction

Osteosarcoma (OS) is the most common malignant bone tumor, occurring predominantly in adolescents, with a secondary peak of incidence after 60 years of age [1, 2]. The characteristics of OS often differ between elderly and younger patients. OS in younger patients primarily affects the knee joint, while secondary neoplasms and axial locations are more common in elderly patients [3, 4]. The standard treatment for OS consists of tumor resection with adequate margins and adjuvant chemotherapy with high-dose methotrexate, doxorubicin, cisplatin, and sometimes ifosfamide (IFO). However, the efficacy of IFO-based adjuvant chemotherapy remains controversial. Although single-arm studies have suggested that adding IFO has beneficial effects in standard responders, randomized controlled trials have failed to show improved survival with the addition of IFO and etoposide in standard responders [5–8]. Additionally, elderly patients are less likely to tolerate intensive chemotherapy, and the role of adjuvant chemotherapy in this group remains controversial [9–12]. Despite these intensive multimodal treatments, approximately 40% of patients experience disease recurrence [13]. Therefore, determining the patient subset expected to benefit from these chemotherapies remains a matter of concern. Recent advances in next-generation sequencing have revealed that OS is characterized by genomic complexity and instability with enrichment for rearrangements and somatic copy number alterations [14, 15]. However, genetic differences between younger and older patients and genetic signatures associated with chemotherapy response are not well understood. Since 2019, cancer genome profiling (CGP) tests have been approved by the public reimbursement system in Japan, facilitating the accumulation of genetic data with clinical information [16]. This study aimed to investigate genetic differences between younger and elderly patients with OS and to identify gene signatures that are correlated with chemotherapy sensitivity using CGP data.

## Patients and methods

Clinical and genetic data for patients were obtained from the Center for Cancer Genomics and Advanced Therapeutics (C-CAT) database on June 20, 2023. Information on the CGP test, age, performance status (PS) at the CGP test enrollment, sex, response to chemotherapy, diagnostic date, date of CGP test enrollment, last follow-up date, and oncological status at the last follow-up date were collected. Patients < 40 years old were defined as younger and those ≥ 40 years old were defined as older. This cutoff age was determined based on the tolerability of intensive chemotherapy, as commonly applied in the eligibility criteria for clinical trials for OS [5, 8]. The response to each regimen, whether monotherapy or combination therapy, was documented by the treating physician according to RECIST criteria. The dataset included 204 patients with OS. Patients with extraskeletal (n = 3), parosteal (n = 2), or periosteal OS (n = 1) were excluded from this study. Reports on CGP tests were available for all patients. All CGP tests were performed using FoundationOne® CDx (Chugai Pharmaceutical Co., Tokyo, Japan; Foundation Medicine Inc., Cambridge, MA, USA), a comprehensive genomic profiling test that analyzes DNA from tumor tissues using a hybrid capture method, covering 324 genes (https://www.rochefoundationmedicine.com/home/services/cdx.html). In this study, variants of uncertain significance were excluded from the analysis. Informed consent was waived because the study was based on C-CAT data, for which patients had already provided consent for research purposes after receiving an explanation from their physician. This study was approved by the Institutional Review Boards and Ethics Committees of Osaka University Hospital and Komagome Hospital (Nos. 21376 and 2819, respectively).

### Clustering analysis

All pathogenic mutations detected using the Foundation One CDx panel were assembled into a binary matrix format for each patient. The dimensions of this input matrix were reduced to two using the Uniform Manifold Approximation and Projection (UMAP) method via the umap package for R, version 0.2.10.0. A clustering analysis was performed using Hierarchical Density-Based Spatial Clustering of Applications with Noise method (HDBSCAN) using the dbscan package for R, version 1.1–12. For each cluster, genetic variants with a higher frequency than those in the other clusters, with an odds ratio of 2 or higher and a significance level of *P* < 0.05, as well as patients who received IFO were evaluated using the Kyoto Encyclopedia of Genes and Genomes (KEGG).

### Statistical analysis

Overall survival was defined as the time from the date of enrollment in the CGP test to death or the last follow-up date, and overall survival rates were assessed using the Kaplan–Meier method. Survival rates were compared using log-rank tests. Fisher’s exact tests were used to compare proportions. All statistical analyses were performed using R software version 4.1.2. *P* < 0.05 indicated statistical significance.

## Results

### Genetic alterations in this cohort

**Fig. 1:**
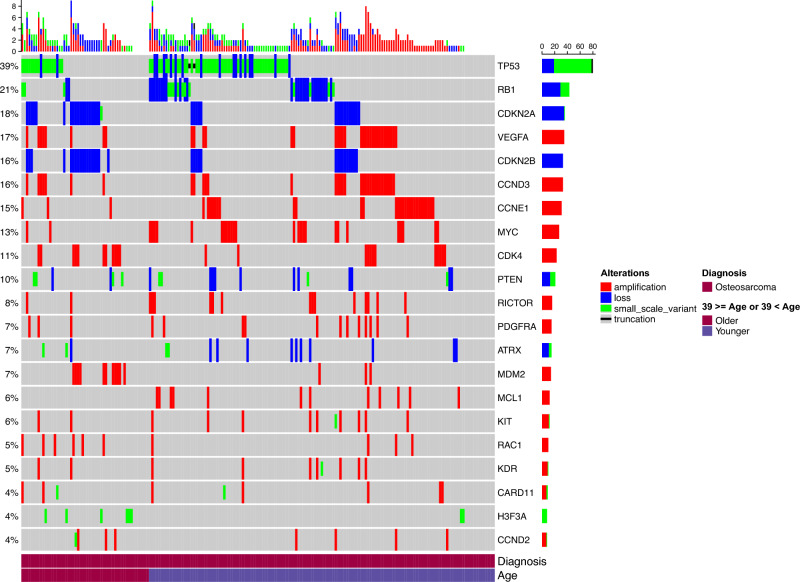
Oncoprint of commonly occurring gene alterations

In total, 204 patients participated in this study, including 122 males and 82 females. The median patient age at diagnosis was 20 years (range 7–80 years). Among the 204 patients, 149 were under 40 years of age and 55 were 40 years or older. The distribution of ECOG performance statuses was as follows: 87 patients had a score of 0, 80 had a score of 1, 16 had a score of 2, 5 had a score of 3, 1 had a score of 4, and 15 patients had an unknown status. The sub-pathological classifications were as follows: 20 patients had osteoblastic OS, 25 had chondroblastic OS, 7 had fibroblastic OS, 4 had high-grade surface OS, 140 had OS NOS, 4 had secondary OS, 1 had small cell OS, and 3 had telangiectatic OS. The most common genetic alteration was *TP53* (79 patients; 39%), followed by *RB1* (42 patients; 21%), *CDKN2A* (36 patients; 18%), *VEGFA* (35 patients; 17%), *CDKN2B* (33 patients; 16%), and *CCND3* (33 patients; 16%) (Fig. 1).

### Age is correlated with oncogenic gene alterations and chemotherapy response

*CCNE1*, *MCL1*, *MYC*, and *RB1* were significantly associated with a younger age, whereas *CDK4*, *CDKN2A*, *CDKN2B*, *H3F3A*, *KMT2D, MDM2*, *RAC1*, and *SETD2* were significantly associated with an older age (Table 1). To verify our results, we utilized the publicly available AACR Project GENIE database (https://genie.cbioportal.org/) [17]. The GENIE cohort included 326 patients with OS, including 232 patients aged 40 years or younger and 94 patients older than 40 years. In this cohort, there were significant correlations between gene alterations and age (Table S1). The gene alterations associated with age common to both cohorts were *CCNE1* in younger patients and *CDKN2A*, *CDKN2B*, and *STED2* in older patients. Next, we analyzed the relationship between age and chemotherapy response and found that older age was significantly correlated with a poor chemotherapy response (Table 2).

**Table 1:** Gene alterations associated with age

	Gene	Odds ratio	*P* value
Younger patients (N = 149)	CCNE1	4.01	0.016
	MCL1	Infinity	0.039
	MYC	5.34	0.018
	RB1	3.3	0.018
Older patients (N = 55)	CDK4	2.85	0.024
	CDKN2A	4.75	< 0.001
	CDKN2B	4.35	< 0.001
	H3F3A	9	0.005
	KMT2D	11.61	0.019
	MDM2	12.17	< 0.001
	RAC1	4.44	0.025
	SETD2	Infinity	0.019

**Table 2:** Chemotherapy response according to age

	Total Number	Ifosfamide		Doxorubicin		Cisplatin		Methotrexate	
		CR/PR	SD/PD	CR/PR	SD/PD	CR/PR	SD/PD	CR/PR	SD/PD
Younger patients	149	29	54	32	64	33	65	25	58
Older patients	55	0	19	2	23	2	18	1	12
Odds ratio		Infinity (2.27-Infinity)		5.69 (1.27–52.78)		4.52 (0.98–42.50)		5.11 (0.69–229.58)	
*P* value		0.001		0.012		0.035		0.175	

### Gene clustering reveals distinct chemotherapy response subsets

**Fig. 2:**
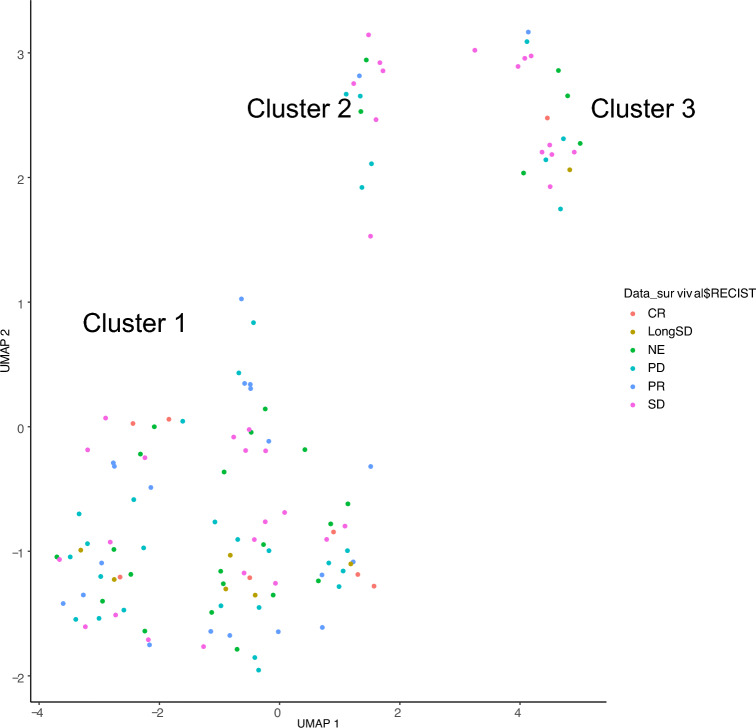
Unsupervised clustering of oncogenic alterations in patients undergoing chemotherapy

**Fig. 3:**
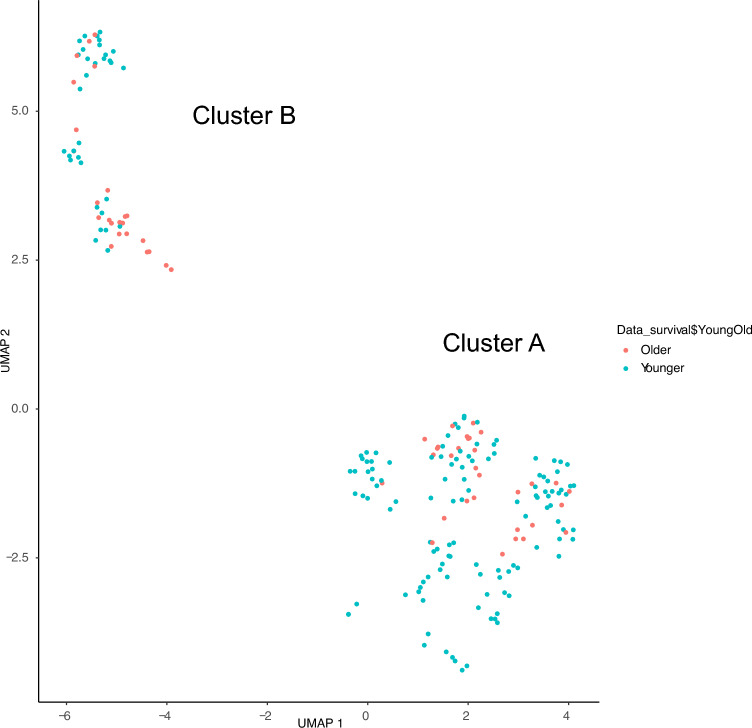
Unsupervised clustering of oncogenic alterations in all patients

We examined gene alterations in 128 patients who received chemotherapy to identify subsets associated with the chemotherapy response. Among these patients, genetic alterations were observed in *TP53* (43 patients), *RB1* (28 patients), *VEGFA* (21 patients), *CCND3* (20 patients), *CDKN2A* (18 patients), and *CDKN2B* (18 patients). Unsupervised clustering revealed three distinct groups (Clusters 1–3). Patients in Cluster 1 showed a significantly better response to the IFO regimen than those of Clusters 2 and 3 (Fig. 2, Table S2). Furthermore, unsupervised clustering of all patients revealed two distinct groups (Clusters A and B). Cluster A tended to show a favorable chemotherapy response (Fig. 3, Table 3). Interestingly, Cluster A harbored highly similar genetic alterations to those in Cluster 1 among the 128 patients who received chemotherapy (Table 4). Younger patients were significantly more likely to be included in Cluster A than in Cluster B (Table S3,* P* < 0.01). Next, we investigated whether the chemotherapy response could be predicted based on shared gene alterations in the two clusters and found that patients who had *TP53*, *RB1*, or *CCNE1* alterations but did not have *CCND3*, *CDK4*, *CDKN2A*, *CDKN2B*, *MDM2*, *PDGFRA*, or *VEGFA* alterations tended to have a better response (Table 5). These results suggest that heterogeneous OS consists of different genetic clusters and that a combination of gene alterations is useful for predicting the chemotherapy-responsive subset of patients with OS.

**Table 3:** Chemotherapy response according to unsupervised clustering

	Total Number	Ifosfamide		Doxorubicin		Cisplatin		Methtorexate	
		CR/PR	SD/PD	CR/PR	SD/PD	CR/PR	SD/PD	CR/PR	SD/PD
Cluster A	95	26	50	29	59	30	59	22	49
Cluster B	33	3	23	5	28	5	24	4	21
Odds ratio		3.94 (1.04–22.40)		2.73 (0.90–10.00)		2.42 (0.80–8.96)		2.34 (0.67–10.48)	
*P* value		0.042		0.069		0.106		0.194

**Table 4:** Gene alterations in each cluster

	Gene alterations	
	Cluster 1	Cluster A
Positive	CCNE1	CCNE1
	RB1	RB1
	TP53	TP53
Negative	CCND3	CCND3
	CDK4	CDK4
	CDKN2A	CDKN2A
	CDKN2B	CDKN2B
	MDM2	MDM2
	PDGFRA	PDGFRA
	VEGFA	VEGFA
	KDR	CCND2
	KIT	
	KRAS

**Table 5:** Chemotherapy response depends on each gene alteration in all patients

	Ifosfamide		Doxorubicin		Cisplatin		Methtorexate	
	CR/PR	SD/PD	CR/PR	SD/PD	CR/PR	SD/PD	CR/PR	SD/PD
Patients with alterations in TP53, RB1, or CCNE1, and without alterations in CCND3, CDK4, CDKN2A, CDKN2B, MDM2, PDGFRA, or VEGFA	19	29	21	36	21	37	15	32
Others	10	44	13	51	14	46	10	26
Odds ratio	2.88 (1.17–7.08)		2.29 (1.02–5.16)		1.86 (0.84–4.16)		1.22 (0.47–3.16)	
*P* value	0.027		0.067		0.16		0.81	

### *MYC* amplification is associated with ifosfamide response

We examined the correlations between gene alterations or KEGG signaling pathways and the response to IFO. We found that *MYC* amplification was significantly associated with the IFO response, and no significant correlation was observed between the KEGG pathway and the IFO response (Table 6 and Table S4). Previous studies have reported that the *MYC* copy number is associated with the response to paclitaxel and mTORC1/2 inhibitors [18, 19]. *MYC* sensitizes cancer cells to mitotic blockers by upregulating pro-apoptotic proteins and suppressing anti-apoptotic proteins [18]. However, the exact mechanism by which *MYC* acts as an IFO sensitizer remains unclear. Further studies are needed to verify this finding and elucidate the underlying mechanism.

**Table 6:** Chemotherapy response for ifosfamide according to gene alteration

		Ifosfamide		Odds ratio	*P* value
		CR/PR	SD/PD		
TP53	+	13	22	1.87 (0.70–4.98)	0.173
	–	16	51		
RB1	+	10	11	2.93 (0.96–8.99)	0.055
	–	19	62		
CDKN2A	+	2	14	0.32 (0.03–1.53)	0.145
	–	27	59		
VEGFA	+	2	14	0.32 (0.03–1.53)	0.145
	–	27	59		
CDKN2B	+	2	13	0.34 (0.04–1.69)	0.221
	–	27	60		
CCND3	+	3	13	0.54 (0.09–2.18)	0.547
	–	26	60		
CCNE1	+	8	10	2.38 (0.71–7.74)	0.147
	–	21	63		
MYC	+	8	6	4.18 (1.13–16.47)	0.021
	–	21	67		
CDK4	+	1	9	0.26 (0.01–2.01)	0.274
	–	28	64		
PTEN	+	1	9	1.50 (0.30–6.53)	0.503
	–	28	64		

### Performance status is correlated with survival

**Fig. 4:**
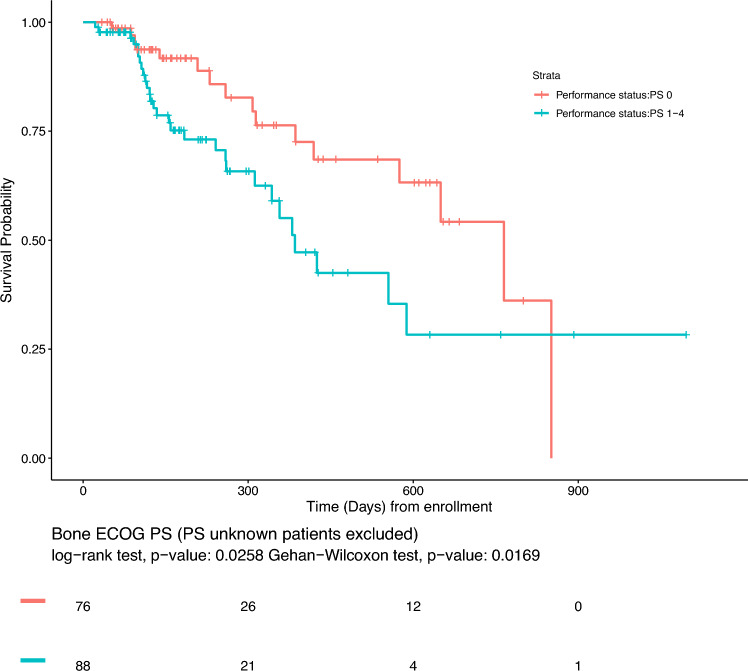
Kaplan–Meier curve for survival after CGP testing based on Performance Status (*P* < 0.05)

To explore the optimal timing for the CGP test, we examined the correlation between PS and survival. We found that patients with a PS of 0 had a better prognosis than that of patients with other PS scores (*P* < 0.05) (Fig. 4). This result suggests that the CGP test should be used before PS reduction to maximize the ability to select optimal treatment options.

## Discussion

OS is the most common, yet rare, bone malignancy in adolescents and young adults, with an incidence of 3.3 per 1000,000 [4]. Owing to its rarity and clinical heterogeneity, it is extremely difficult to build firm evidence based on clinical trials. Therefore, genetic analyses of OS are fundamental to deepening our understanding of this disease, developing treatment strategies, and improving clinical outcomes. However, whole-exome sequencing and multi-omics analyses are still not always feasible, and CGP is relatively widely available in outpatient clinics.

In this study, CGP data revealed that *CCNE1*, *MCL1*, *MYC*, *RB1, CDK4*, *CDKN2A*/*B*, *H3F3A*, *KMT2D, MDM2*, *RAC1*, and *SETD2* mutations are associated with age (Table 1). *CCNE1*, *CDKN2A*, *CDKN2B*, and *STED2* were also associated with age in the AACR Project GENIE cohort. A large array-based next-generation sequencing study has demonstrated that copy number variants were more frequent in *CCND3*, *AURKB*, *CCNE1*, *GID4,* and *MYC* in younger patients with OS, whereas *MDM2*, *CDKN2A/B*, and *FRS2* were more frequently altered in older patients with OS [20]. Although the cutoff ages differed (40 and 30 years), both studies revealed that *CCNE1* and *MYC* amplifications occurred predominantly in younger patients, whereas *MDM2* amplification and *CDKN2A/B* deletions occurred in elderly patients. In previous studies, *CCNE1* and *MYC* were amplified in 7–33% and 7–52% of OS samples, respectively [21–24], and co-amplification of *CCNE1* and *MYC* was a rare event (1.1%) with an aggressive clinical course [25]. *MDM2* was amplified in 7–26% and *CDKN2A* was deleted in 7–25% of OS samples [21–24]. In contrast, two studies using cutoff ages of 18 and 21 years showed no genomic differences between younger and older age groups [21, 22]. These differences among studies may be due to the different cutoff ages and the relatively small number of patients. Consistent with our speculations, one study showed that OS patients < 18 years of age have significantly more clustered rearrangements associated with chromothriptic regions than patients ≥ 50 years of age. However, no statistical difference was observed between patients aged < 18 and those aged 18 to 50 [26]. Taken together, these findings suggest that heterogeneous OS develops through multiple distinct oncogenic mechanisms and that these mechanisms are, to some extent, age-related.

Several drug resistance factors, such as ABC transporters, DNA repair factors, non-coding RNA, and cancer stem cells, have been reported in OS [27]. RNA sequencing data for 43 primary OS samples from the TARGET-OS database revealed that chemoresistant OS is characterized by the upregulation of osteogenic markers and downregulation of immune response markers [28]. Two genomic sequencing studies involving 25 and 48 samples have shown that chemo-responders have higher frequencies of COSMIC3 signatures associated with homologous recombination repair deficiency than those of non-responders [26, 29]. Although these three studies compared chemotherapy responses based on histological evaluations, we did not have histological data and employed reported data based on the RECIST criteria for each chemotherapy regimen. Owing to the targeted sequencing procedure and differences in methods for assessing chemosensitivity, we were unable to identify osteogenic, immune-related, or DNA repair markers as chemosensitivity indicators. However, gene alteration signatures, based on unsupervised gene clustering, tended to reflect the chemotherapy response. Previous studies have shown that *MDM2* amplification is mutually exclusive of *TP53* alterations [21, 22]. Additionally, an unsupervised clustering analysis has shown that *TP53*, *MDM2*-*CDK4*, and *CDKN2A/B* alterations form distinct groups [30]. Consistent with these previous findings, our unsupervised clustering revealed distinct *CCNE1* and *TP53* alteration-positive and *MDM2* and *CDKN2A/B* alteration-positive groups, with different age distributions. Therefore, the chemotherapy-responsive subset characterized by these distinct gene alteration signatures may be generalizable to the OS population. We also found that *MYC* amplification was significantly correlated with the IFO response (Table 6). Several studies have reported that *MYC* amplification is associated with cisplatin and methotrexate resistance and a poor prognosis [24, 31, 32]. This discrepancy in responses to IFO and other drugs has led to controversial results in clinical trials [5–8]. Notably, in this study, age showed the strongest correlation with chemotherapy response. Additionally, the gene alteration signature and *MYC* amplification associated with chemotherapy response were significantly associated with age. These results suggest that age-biased gene alterations may contribute to the high chemotherapy sensitivity observed in young patients with OS. Further studies are needed to validate our findings and to explore the utility of the CGP test in selecting a chemotherapy regimen.

In Japan, the CGP test is covered by public insurance for patients with advanced disease after the completion of standard treatment. However, the CGP test serves various purposes; it is used for diagnosis, prognostic prediction, and the identification of cancer predisposition, in addition to guiding genome-informed therapies. Therefore, the optimal timing for testing must be determined by a specialist based on the specific disease context. Clinical practice guidelines recommend that the timing of the CGP test should not be determined solely by the line of treatment [33]. Earlier CGP testing may improve access to genotype-matched clinical trials because previous treatment lines sometimes impede eligibility [34]. Furthermore, a decline in overall health is a common reason for not undergoing genotype-matched therapy [35]. Our results also suggest that earlier CGP testing may contribute to favorable outcomes. The accumulation of evidence from CGP testing, beyond the selection of genome-matched therapy, may lead to the earlier adoption of CGP tests in the future, especially in rare cancers.

This study had several limitations. First, this study had an inherent selection bias, as it consisted mainly of patients with poor prognoses because the CGP test is indicated for advanced cases. Second, we used the FoundationOne® CDx; accordingly, our analyses were limited to 324 genes related to oncogenesis. Therefore, we did not investigate gene alterations related to other factors, such as osteogenesis or immunosuppression. Third, with respect to chemotherapy sensitivity, a central review was not conducted, and responses were assessed by each treating physician. Additionally, owing to the lack of dose intensity data for each regimen in the C-CAT database, we were unable to evaluate chemotherapy sensitivity in relation to the intensity of chemotherapy. Furthermore, we found a relatively weak correlation between gene clustering and response to chemotherapy. Therefore, further studies using CGP tests that encompass a broader range of gene alterations are necessary to identify robust predictors of chemotherapy sensitivity. Despite these limitations, the relatively large patient cohort and the use of a readily available CGP test are key strengths of this study.

In conclusion, our analysis of CGP data revealed distinct age-related distributions of genetic alterations in patients with OS. The combination of these gene alterations may be valuable for predicting sensitivity to chemotherapy. Early CGP testing could be beneficial in selecting an optimal treatment strategy.

## Supplementary Information

Below is the link to the electronic supplementary material.

Supplementary file1 (XLSX 14 KB)

## Data Availability

Raw data for this study were obtained from C-CAT Research-Use Portal (https://www.ncc.go.jp/en/c_cat/use/index.html). The data are accessible after institutional review at your institution and approval from the C-CAT data utilization committee. Derived data supporting the conclusions of this study can be obtained from the corresponding author upon reasonable request.
